# A systems approach toward climate resilient livelihoods: A case study in Thai Nguyen province, Vietnam

**DOI:** 10.1016/j.heliyon.2020.e05541

**Published:** 2020-11-18

**Authors:** Tuan M. Ha, Insa Kühling, Dieter Trautz

**Affiliations:** a Thai Nguyen University of Agriculture and Forestry, Thai Nguyen City, Viet Nam; b Kiel University, Kiel, Germany; c Osnabrück University of Applied Sciences, Osnabrück, Germany

**Keywords:** Social science, Climate change adaptation, Climate-smart agriculture, System dynamic modelling, Bayesian belief network, Systemic interventions

## Abstract

This study aims to identify strategic actions towards climate resilient livelihoods and secure income for smallholder farmers in Thai Nguyen province of Vietnam using a systems approach and system dynamic modelling tools. Information and data for this research was collected through surveys, interviews, focus group discussions and workshops with relevant stakeholders and 187 farmers in two vulnerable districts during October 2019–April 2020. Findings of this study uncovered a number of shortcomings of the government policies and approaches in climate change adaptation. Local initiatives, community learning and ownership seem to be neglected. This research has substantiated the effectiveness and validity of systems approaches and tools in structuring and solving complex issues in agricultural research and development under the interwoven relationships between environmental and human factors. Climate resilient production models and practices are just part of the systemic interventions that need to be implemented in a coordinated manner towards a more resilient future of the farming communities. This study has addressed the current knowledge gap and the need for using integrated approaches and decision support systems for unravelling ill-structured and/or complex issues of climate change adaptation (CCA). It also provided practical recommendations for informed CCA policies and implementation.

## 1 Introduction

The growing global population together with the ongoing loss of arable soils lead to an increasing demand for agricultural production (Tilman et al., 2011). Together with climate change, this causes new challenges for agricultural systems worldwide (Azadi et al., 2019; Foley et al., 2011). To cope with these changing boundary conditions, agricultural systems of the future need to be shaped sustainably. This pathway of “Sustainable Intensification” includes the maintenance of agricultural production while minimizing environmental damage (Baulcombe et al., 2009). Possible implementation strategies vary depending on the region. In developing countries like Vietnam, agriculture is an important contributing factor to economic growth and poverty alleviation, particularly via providing food and employment opportunities. Nonetheless, agriculture is not always the means for poverty alleviation due to both economic and natural risks (Cuong, 2011; Ha, 2016).

Climate change induces considerable impacts on global agricultural production (Adedoyin et al., 2020; Anwar et al., 2013; Nechifor and Winning, 2019; Zabel et al., 2014), causing pressure on the world's food supply system and food security in many parts of the world (Gebru et al., 2020; Kurukulasuriya and Rosenthal, 2013; Rahut and Ali, 2018). This is because agriculture is greatly dependent on climate change and variability (Parry et al., 2007; Sarker and Rashid, 2012). Especially, smallholder farmers and poor communities in developing countries are more vulnerable to the impact of climate change (Ancog et al., 2019; Frimpong et al., 2020; Kurukulasuriya and Rosenthal, 2013; Marie et al., 2020; Sarker and Rashid, 2012). As such, there is a high need for adaptation strategies (Adzawla et al., 2019; Aniah et al., 2019; Idrissou et al., 2020; Muema et al., 2018), particularly ecosystem-based adaptation (Maina et al., 2020) and/or area-specific adaptation measures (Marie et al., 2020).

With 65.6% of the population residing in rural areas (GSO, 2019b), livelihoods of the rural population in Vietnam are mainly reliant on agricultural production (Timler et al., 2020). Its agricultural system is characterized by small-scale rice-based production and land fragmentation (Ha et al., 2017). Vietnam is amongst the top five countries that are worst affected by the impact of climate change (World Bank, 2011). According to Chaudhry and Ruysschaert (2008), climate change creates a real threat to the country's socio-economic development. Its increased severity would result in diminished crop production and food security, disrupted and reduced water supplies, increased risks of forest fires and energy security, etc (USAID, 2011). The rural poor, especially ethnic minorities in the northern mountainous region, are among the most vulnerable groups (Son et al., 2019; USAID, 2011). Vulnerability is closely related to livelihood insecurity and poverty. In spite of some localized successes, smallholder farmers are most affected by climate change due to their limited information and/or financial and technical assistance (Chaudhry and Ruysschaert, 2008).

According to Ensor and Harvey (2015), climate change adaptation (CCA) is a “complex issue” due to the interplays among social and environmental challenges. The topic attracts high attention in both research and practice. Lotz-Sisitka et al. (2015) asserted that sustainability concerns are most often described as “wicked problems” and/or “nexus issues” (i*.e. the causal interrelationships among climate change, energy, water resources, food security, and livelihoods, etc.*) which are characterized by high levels of complexity, ambiguity, controversy and uncertainty both with regard to what is going on and concerning what needs to be done. This is consistent with Hertel and Rosch (2010) who stated that climate change is a “wicked problem” since it does not have any clear-cut solution. Therefore, the authors suggested the need for simulation tools to understand and deal with such complex issue. Additionally, according to Wals and Schwarzin (2012), to address the complex issue such as climate change adaptation, it is necessary to employ systems thinking and systems approaches. Other studies also highlighted a strong need for adopting an integrated approach and decision support/modelling tools in climate change adaptation research to cope with complexity and uncertainty in decision making (Karner et al., 2019; Phan et al., 2020; Sperotto et al., 2017; Terzi et al., 2019; Wenkel et al., 2013).

The methodology and systems approaches have been proven the most suitable and effective ones in structuring and solving complex problems for developing appropriate management strategies and systemic interventions toward sustainability (Bosch et al., 2013b; Ha, 2016). In contrast to the traditional approach, which is often called reductionist approach with linear thinking in solving problems, a systems thinking approach employs a holistic outlook on the multi-aspects and interrelationships of complex issues (Bosch et al., 2013a; Rubenstein-Montano et al., 2001; Sterman, 2001). It helps discover the hidden causes under multi-dimensional contexts (Maani, 2013), making systemic interventions possible for sustainable outcomes (Bosch et al., 2013a).

Nonetheless, recent reviews by Sperotto et al. (2017) and Terzi et al. (2019) reveals limited research on holistic assessment of climate change impacts and development of CCA strategies. Particularly, there is a dearth of research using systems approaches and system dynamic modelling tools for identifying strategies and interventions towards climate resilient livelihoods. Our approach is novel in its holistic approach through using an established systems-based Evolutionary Learning Laboratory (ELLab) framework. Its built-in user-friendly system dynamic modelling tools enabled local stakeholders and the research team to understand key challenges and interrelationships among various factors that together influence the lives of the smallholder farmers. It also helped to define relevant strategies toward climate resilient livelihoods.

Therefore, this paper aims to understand the current challenges of smallholder farmers and identify the most locally appropriate interventions toward sustainable livelihoods and income for the farming households in Thai Nguyen province of Vietnam by using the systems approach. Particularly, relevant information and data were collected and analysed to understand their current livelihoods situations and challenges, responses of the local government and other actors. Workshops and focus group discussions were then organised with relevant stakeholders to develop a systems model of the current situation using causal loop diagram modelling, and identify systemic interventions using Bayesian network modelling. This study contributes to the literature through employing system dynamic modelling tools in climate change impact assessment and development of strategies toward climate resilience; and providing informed recommendations to guide CCA policies and implementation.

## 2 Approach and methodology

### 2.1 Research locations

The study was conducted in Thai Nguyen, one of the provinces in the northern midland and mountainous region of Vietnam. The province has a total natural land area of 3,562.82 km^2^ (MPI, 2019). As of 1 April 2019, the province had a total population of 1.29 million residents with 29.9% of ethnic minorities and 68.1% people living in rural areas (GSO, 2019a). Its topography is characterized by hills and mountains interspersed with fields (Thai Nguyen PPC, 2016). Agricultural land makes up 23% of the total natural land area (VUFO, 2018). Mountainous and hilly areas account for 48.4% and 31.4%, respectively (World Bank, 2005). There are two distinctive seasons, namely, hot and rainy (May–October) and cool and dry (November–April) seasons. The rainfall is between 1,400 and 2,700mm (Hien et al., 2016), in which rainfall in the rainy season contributes to 75–80% of the total annual precipitation. July and August have the highest precipitation rate with over 300 mm/month (Thai et al., 2017). Average temperature is around 23–24 °C (World Bank, 2005). The lowest and highest temperature have been ever recorded at 1 °C and 40.7 °C, respectively (World Bank, 2018). The province has been significantly affected by the impacts of climate change such as storms, flood and soil erosion during rainy seasons, and water shortage and drought during dry seasons (Thai et al., 2017).

**Figure 1:**
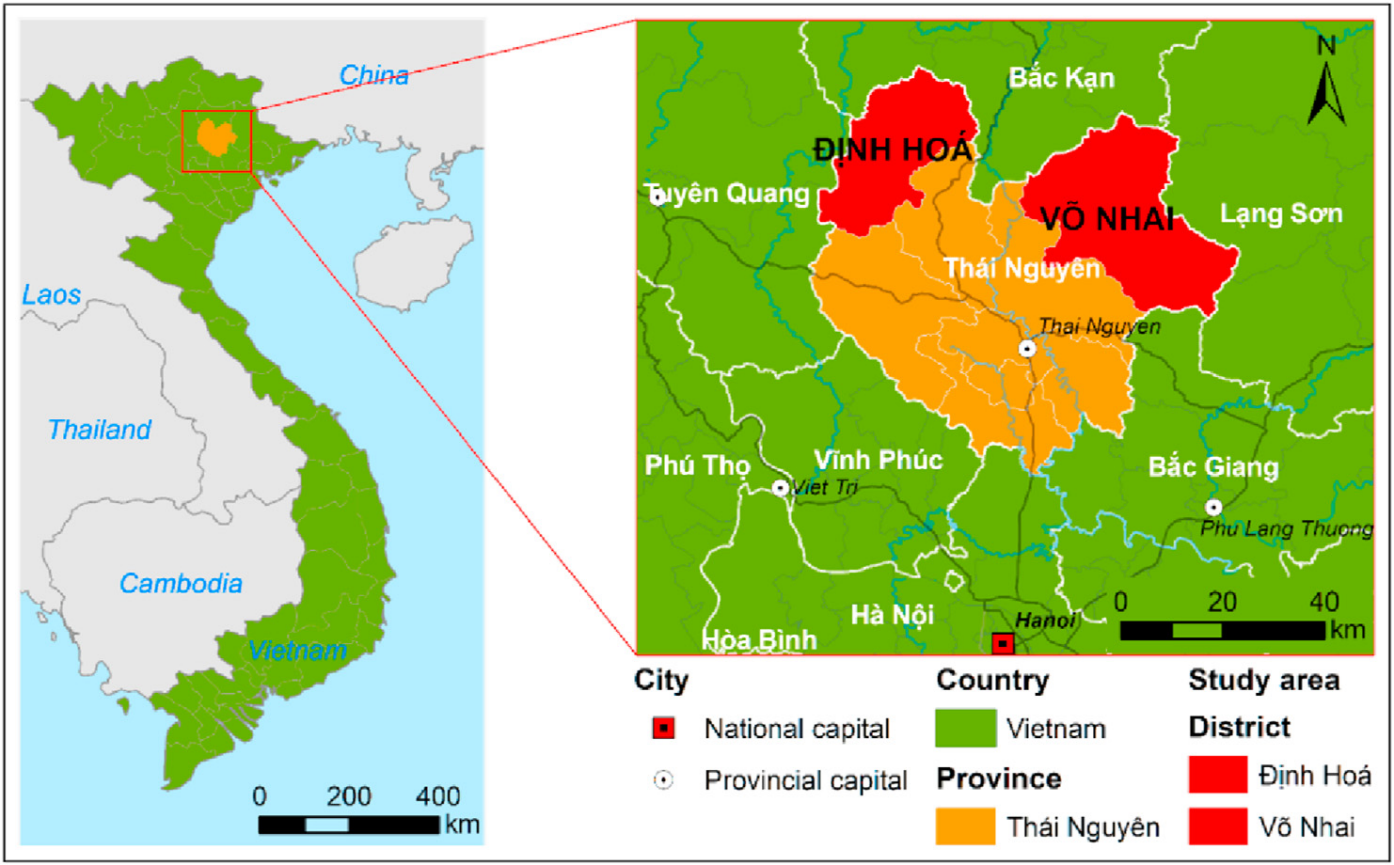
GIS map of the research locations (red stars on the map).

Two northern mountainous districts of Thai Nguyen, namely, Dinh Hoa and Vo Nhai, were selected for this study (Figure 1). Due to increased number of hot days, heavy rain, hail, storms, flood and landslide during rainy seasons, and water shortage, drought and cold spells during dry seasons (Dinh Hoa DPC, 2019; Vo Nhai DPC, 2019) these two poor districts are highly vulnerable to climate change.

### 2.2 Methodology

**Figure 2:**
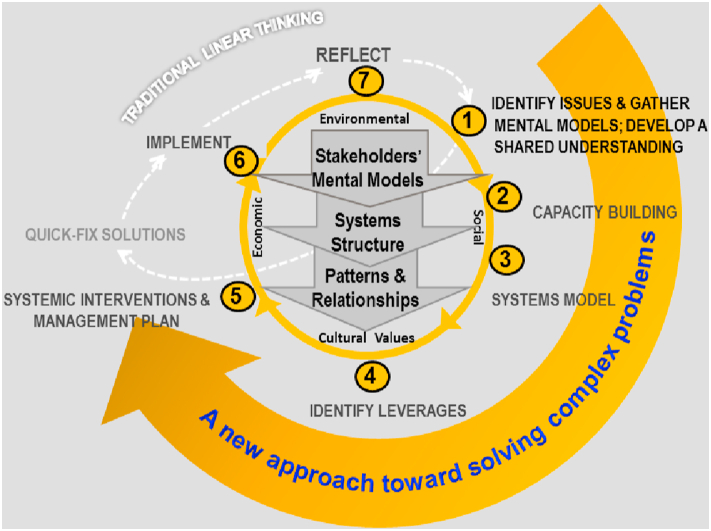
Evolutionary Learning Laboratory for Managing Complex Issues under conditions of interwoven relationships between economic, social, environmental and cultural factors (Source: modified from Bosch et al., 2013b).

This study employed a participatory approach through combining field surveys, focus group discussions (FGD) and workshops during October 2019–April 2020 to gather qualitative and quantitative information and data from the studied locations. The first five Steps of the Evolutionary Learning Lab (ELLab) (Figure 2) were used for this research. Detailed description of all the steps of the ELLab can be seen in Ha et al. (2016).

Step 1: Issue identification

This step comprised the following activities:

*Desktop studies and key informant interviews*.

The purpose of this step was to collect and analyse relevant information on the current situation of agricultural production, key challenges, and impacts of climate change on the local livelihoods; policies and measures for climate change adaptation in the research locations.

The key informants were comprised of representatives of the local governments from provincial to commune levels, and relevant organisations, including Department of Agriculture and Rural Development (DARD), Extension Centre, District Centres for Agricultural Services (2 persons per organisations), leaders of commune authorities and extension staff (8 people at 4 communes), and local civil organisations (including women unions, farmers’ associations and youth unions) (2 people per organisation).

Existing documents and data were collected from the above mentioned organisations and via literature review.

*Household survey*.

Dinh Hoa and Vo Nhai districts were determined and selected for this study based on the above activity. The purpose of this survey was to gather information on their livelihoods, key challenges and needs, impacts of climate change, adaptation measures and/or initiatives, and potential climate resilient livelihoods.

In each district, two most vulnerable communes were selected for the household survey with a sample size of 39–56 farmers/commune. A stratified sampling method was used to ensure the representativeness of respondents with regard to geographical locations (villages that are vulnerable to the impacts of climate change), gender, age, wealth groups, ethnicity, and types of production systems (see Table 1).

**Table 1:** Profile of the surveyed households (n = 187).

Category	Statistics
Age (years old)	
Mean	45.22
*S.E*	*0.821*
Gender (%)	
Male	41.7
Female	58.3
Location (number of respondents)	
Dinh Hoa district	95
Vo Nhai district	92
*Total*	*187*
Wealth groups (%)	
Above average	4.3
Average	75.4
Marginally poor	12.8
Poor	7.5
Household size	
Mean	4.51
*S.E*	*0.100*
Number of main labourers/household	
Mean	2.61
*S.E*	*0.078*
Number of members working in the agriculture & forestry sector	
Mean	2.05
*S.E*	*0.090*
Number of members working in other sectors	
Mean	0.66
S.E	*0.062*

*Workshops and focus group discussions for identifying the most potential livelihood models and production practices*.

After the personal interviews, the same interviewed respondents at district and commune levels together with representative farmers were invited for discussion workshops (one workshop of 50 people per district). Small focus group discussions (FGD) and plenary discussions were organized to discuss (1) key challenges in agricultural production and livelihoods; (2) reasons for the challenges; (3) solutions and/or recommendations to the local government for addressing the challenges. After that, participants were facilitated to discuss and identify the most potential climate resilient livelihood models and production practices in the localities under the context of climate change.

Step 2: Capacity building

After the above Steps, the research team identified and selected 15 key participants for a mini-workshop to consolidate and analyse information for both districts. The participants include representatives from district departments of agriculture and rural development, centres for agricultural services, commune leaders & extension staff, and experienced farmers from both districts. A training workshop was followed to build capacity of the key participants on understanding and identifying patterns of causal relationships among different factors that influence livelihoods and income of the local farmers.

Steps 3–5: Developing a systems model and identifying levers and systemic interventions

A FGD was organized for the key participants and the research team to discuss and develop a systems model (causal loop diagram) of the current livelihood situation of the farmers in Thai Nguyen using Vensim® software (Ventana®, 2011). The systems model depicts causal links among various factors and their interplays that influence livelihoods and income of the target group (local farmers). The model was then used as an input for an extended workshop with wider participation of 50 participants who represent functional departments, extension networks, commune authorities, local community organizations and representative farmers in the two districts.

**Figure 3:**
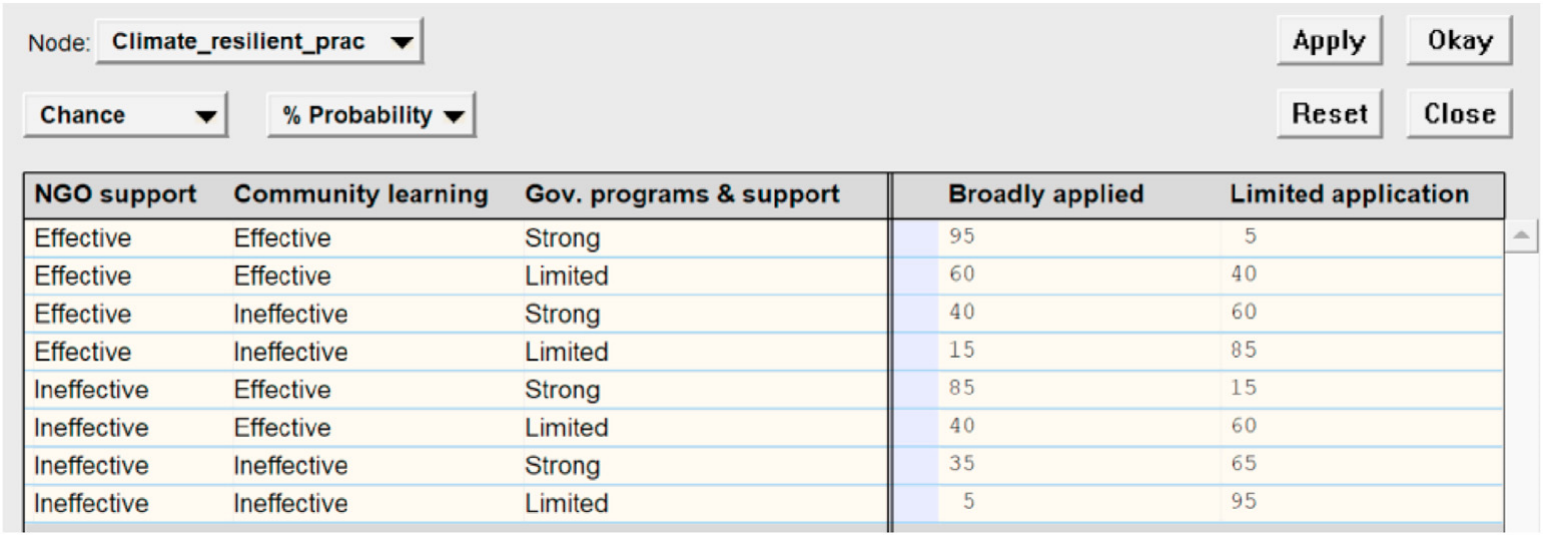
Example of sensitivity analysis using Bayesian network modelling software (translated from Vietnamese).

Comments and feedback from the workshop was used by the research team and the key participants to further revise and validate information in the systems model. Based on the validated causal loop diagram model, the key participants were facilitated to develop a directed acyclic diagram and populate conditional probability tables in Bayesian networks using Netica™ software/decision support tool (Norsys, 2013) with improved household income as a final goal to be achieved. The level of impacts among the variables defined in the systems model were discussed and quantified by the key participants and the research team (see an example in Figure 3).

Future scenario testing was conducted through testing of different states of the variables within the system (Bayesian network model) to identify key leverage points for systemic interventions - “the points of power and/or places within a complex system where small shift in one thing can produce big changes in everything” (Meadows, 1999). The identified levers were those with largest impact on achieving the end goal (household income). The same process was conducted by the key participants to define systemic interventions (those with high impact level towards achieving the identified objectives (key leverage points) and the end goal). Groups of systemic interventions were then discussed to formulate integrated strategies toward climate resilient livelihoods and income for the smallholder farmers in the studied areas.

Model validation: Two workshops were organised afterward with 50 and 70 participants, respectively (one for the two studied districts and one extended workshop at the provincial level with participation of larger stakeholders from provincial and commune levels) to receive feedback and suggestions on the model, impact analysis and strategic interventions. Particularly, the participants were first asked to provide feedback and validate the causal links among all the variables within the systems model. This was followed by their discussion and validation of the conditional probabilities between different states of the parent and child nodes within the Bayesian network model. Finally, the participants were asked to provide inputs and feedback on future scenarios via testing of different interventions and their respective levels of impact on the end goal were agreed by the majority of the participants.

#### 2.2.1 Data analysis

Euantitative data from the household surveys were subject to statistical analysis using Descriptive Statistics, Independent Sample T-Test for equality of means, and Pearson Chi-Square Tests in SPSS software (version 20) (Nie et al., 2011).

#### 2.2.2 Ethical approval

Research design and tools were submitted and approved by the ethical committee of Thai Nguyen University of Agriculture and Forestry prior to the research implementation.

## 3 Results and discussions

### 3.1 Brief overview of recent climatic condition and impacts of climate change in the studied locations

**Figure 4:**
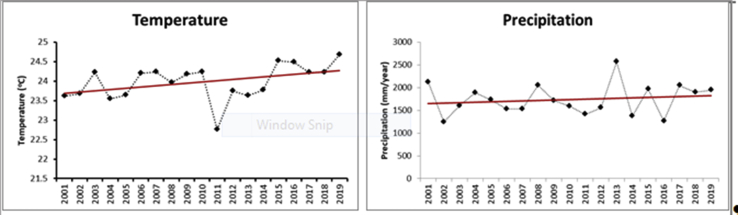
Historical changes of key climatic data in Thai Nguyen province. Source: calculated based on the monthly and annual average data collected from the Meteorological and hydrological station in Thai Nguyen.

Historical data for the period 2011–2019 collected from the meteorological and hydrological station in Thai Nguyen showed an increasing trend of both temperature and rainfall (Figure 4). This reveals an evident change of the local climate in recent years.

Although there was an increase of precipitation during the period, the amount of rainfall was more focused during the rainy season with around 75–80% of the total annual rainfall (Thai et al., 2017), while drought has been more frequent in the dry season.

Table 2 shows considerable influences of climate change on the local residents and agricultural production in Thai Nguyen province. The events reported were mainly natural disasters in rainy seasons, causing considerable losses of lives and properties, particularly agricultural production. Due to the requirements of the provincial government, local communes and districts have to provide annual reports on the damages caused by natural disasters, which mainly occur in rainy seasons. Therefore, losses and damages caused by drought in dry seasons were not part of the annual reports.

**Table 2:** Impacts of climate change in Thai Nguyen in recent 3 years collected from the survey in 2019.

Year	Key events	Impacts
2017	Two major storms (number 2 (Talas) and number 6 (Hato); 1 tropical low pressure event; 8 thunderstorms, tornadoes and landslides; 15 flooding events.	11 deaths reported (increased by 5 people compared to 2016); 917 houses were unroofed; 635 houses were flooded, 335 houses were affected by landslides, fallen trees and lightning strikes; nearly 2,000 ha of rice and vegetables were damaged; over 30,000 cattle and poultry died and swept away, etc. Estimated damage was over VND 160 billion (7 million USD) (increased by 2.2 times compared to 2016).
2018	5 heat waves, 7 moderate rainfalls, heavy rains with lightning and 3 storms, one tropical low pressure and 8 natural disasters.	3 people died and 5 injured; more than 500 houses, 13 schools and 5 cultural houses damaged; over 464 ha of rice, 128ha of annual crops, 24ha of ponds and lakes for aquaculture are affected. Estimated total loss of assets was over 20.7 billion VND (900,000 USD).
2019	10 large-scale heat waves, 7 moderate rainfalls, heavy rains with lightning storms, 3 storms and 13 natural disasters.	7 persons died and 8 injured; 4,800 houses, over 240 electricity poles broken; 57 schools affected; over 860ha of rice, nearly 470ha of vegetables and 420ha of forest affected, etc. Estimated total loss of assets was over 108 billion VND (4.7 million USD).

Dinh Hoa and Vo Nhai were among the most affected districts in the province. For instance, in Dinh Hoa district, five deaths were reported due to flooding; 518 households were seriously damaged by storms and flooding; 714ha of rice and crops, 44ha of forests, 189ha of aquaculture, 11,224 cattle and poultry, and other production infrastructure works were damaged and lost in 2017 with the total damage amount of about 6.52 million USD. In Vo Nhai district, the estimate cost of damages of roads, agriculture and other properties was about 280,900 USD in 2018 due to landslide, storms, flooding.

### 3.2 Livelihood situation and climate change impacts on local farmers

Similar to results of other studies on the smallholder farmers in the northern midland and mountainous region of Vietnam (e.g. Ha et al., 2017; Nguyen, 2017; Son et al., 2019; Timler et al., 2020), the local households in rural districts of Thai Nguyen are mainly reliant on agriculture-based livelihoods. Crop production and animal husbandry account for 62.6% and 15.2%, respectively, of total household income sources. Other off-farm jobs, particularly at industrial zones, were reported as an emerging important source of income in recent years(see Table 3).

**Table 3:** Characteristics of farming households in the studied areas (n = 187).

	Thai Nguyen		Dinh Hoa district		Vo Nhai district		P-value
	Mean	S.E	Mean	S.E	Mean	S.E	
Income sources (%)							
Crop production	62.65	1.920	60.58	2.798	64.78	2.621	0.275
Animal husbandry	15.21	1.260	14.95	1.450	15.49	2.085	0.830
Aquaculture	1.50	0.427	1.79	0.713	1.20	0.460	0.488
Forestry	7.64	0.861	8.14	1.239	7.12	1.198	0.556
Services	2.57	0.691	2.58	0.885	2.55	1.072	0.986
Other sources	10.22	1.469	11.55	1.925	8.86	2.229	0.361
Production area (m^2^)							
Agricultural prod. area	2,969.64	205.305	2,818.59	205.792	3,129.09	362.428	0.451
Forest land area	2,576.61	548.279	2,711.28	960.713	2,434.44	498.074	0.802
Livestock & aquaculture							
Buffalo number	0.20	0.070	0.12	0.058	0.29	0.130	0.227
Cow number	0.37	0.107	0.35	0.129	0.38	0.175	0.863
Pig number	2.74	0.624	3.91	1.075	1.52	0.589	0.056
Poultry number	73.83	11.827	60.20	13.219	88.07	19.817	0.240
Other livestock	0.37	0.184	0.29	0.155	0.44	0.341	0.685
Volume of fish produced (kg/year)	200.00	56.766	154.84	60.682	480.00	134.825	0.047∗
% of fish sold to markets	34.57	8.591	35.00	12.116	34.00	12.667	0.956

Note: S.E: Standard Error of Mean; Independent Sample T-Tests for equality of means were used to compare the indicators between the two districts. ∗P < 0.05.

The production areas and number of livestock reflect the nature of small-scale production in the studied areas. Due to the recent African swine fever since early March 2019, the number of pigs per household was much smaller than that of the previous year. As of 30 November 2019 in Thai Nguyen province, the total number of pigs infected with the fever that had been destroyed was 158,773. In Dinh Hoa district, 15,000 pigs (about 34% of the total number of pig heads in the district) were destroyed by the end of November 2019. Whereas, in Vo Nhai district, there were 10,436 infected pigs were destroyed by 15 October 2019.

Aquaculture is not a major livelihood of the farming households in the studied districts due to the fact that the province locates in the northern midland and mountainous region (MPI, 2019; World Bank, 2005). The volume of fish produced and percentage of fish sold to markets reflect the form of subsistence production of local farmers in both districts. This is consistent with findings of Pucher (2014) in Son La province in northern mountainous region of Vietnam who stated that “pond aquaculture is still performed mainly for subsistence and to supply local markets”.

Table 4 reveals significant impacts of climate change on the smallholder farmers in both districts. Reduced crop and/or livestock productivity, crop losses, and reduced production land and number of crop seasons per year were mentioned as the most important impacts among other. These are followed by frequent power outage due to storms.

**Table 4:** Direct impacts of climate change on the local farming households (household survey, n = 187).

#	Direct impacts on households	Average for Thai Nguyen	By district		P-value
			Dinh Hoa	Vo Nhai	
1	Reduced crop and/or livestock productivity	58.3%	55.8%	60.9%	0.289
2	Crop losses	56.1%	51.6%	60.9%	0.129
3	Reduced production land and number of crop seasons/year	44.9%	45.3%	44.6%	0.520
4	More frequent power cut	36.9%	35.8%	38.0%	0.433
5	Had to change livelihoods	14.4%	7.4%	21.7%	0.004∗∗
6	Changes of land use purposes or crop types	14.4%	6.3%	22.8%	0.001∗∗
7	Water shortage in aquaculture	8.6%	9.5%	7.6%	0.424
8	Other	3.2%	2.1%	4.3%	0.326

*Note:* Pearson Chi-Square Test: ∗∗P < 0.01.

The households in Vo Nhai district were more influenced by climate change with 21.7% of the affected households that their members had to change their livelihoods. Additionally, farmers in Vo Nhai district seem more active in changing land use purposes and crop types in adapting to the changing environment (Table 4).

Results of the key informant interviews showed increasing impacts of climate change in recent years. In Dinh Hoa district, there has been an increasing trend in the occurrence and intensity of storms, heavy rain, landslide and flood in rainy seasons, and increased drought and cold spells in dry seasons. Similar trend was also reported in Vo Nhai district, except for landslide. For instance, in Dinh Hoa, 376.7ha and 20ha of rice and maize, respectively, were damaged by heavy rain and flooding in 2017. In the same year in Vo Nhai, more than 1,000ha and 300ha of maize and rice, respectively, were severely impacted by drought. A significant proportion of households have therefore shifted from rice and maize to fruit crop production as a way of adaptation. These findings are consistent with results from previous studies in Thai Nguyen (e.g. Hien et al., 2016; Hong and Yabe, 2017; Thai et al., 2017). Hien et al. (2016) projected more heavy rainfall during rainy seasons. Particularly, the maximum change of rainfall in July will be 10.6% in 2050, while the precipitation between January–March will decrease by up to 20% by 2050. This implies increased drought and water shortage during dry seasons, while more intensified rain and thus flooding and other related events.

### 3.3 Responses of the local government and other actors

The government of Vietnam has been rather active in developing a range of strategies and action plans in response to climate change as reviewed by ADB (2013) and Nguyen et al. (2017b). Some key responses related to the agriculture, forestry and fishery sector are summarized in Annex 1.

At the provincial level, following the policies and guidance from the central government, the local government of Thai Nguyen has promulgated a number of strategies and action plans in response to climate change. Those efforts have been indicated through Decision No. 1013/QD-UBND (2012) of the local government on approving an action plan for climate change response in Thai Nguyen province, followed by Decision No. 2270/QD-UBND (2016) on climate change prevention and response for the period 2016–2020. The guiding actions and activities include: identifying solutions and projects for mainstreaming climate change response into the provincial development strategy and plans; strengthening organisation capacity, institutions and policies on climate change; raising public awareness and responsibilities; upgrading infrastructure in response to climate change; implementing activities on search and rescue; supporting household relocation; and recovering infrastructure for production, etc. Additionally, the local government issued Decision No. 4157/QD-UBND (2017) on guiding the implementation of support policy and levels of support to local citizens, particularly farming households, for damages caused by climate change and epidemics.

Besides, other agriculture and rural development programs of the province have also contributed to the on-going efforts in adapting to the changing environment in Thai Nguyen. For instance, the national target program on “new rural development” for the period 2010–2020 in Thai Nguyen has provided financial support for upgrading and building more than 420km of irrigation canals (Thai Nguyen PPC, 2019). Climate change adaptation and responses have also been integrated into the socio-economic development plan of Thai Nguyen (period 2015–2020) (Decision No. 260/QD-TTg (2015) by the PM, the agricultural restructuring plan for the period 2017–2020 of Thai Nguyen People's Committee (Decision No. 2018/QD-UBND on 5 July 2017), and annual agricultural production plans of the province. Some typical activities include: upgrading dykes and irrigation systems; building artificial lakes for storing water; forest protection and payment for environmental services; changing of cropping patterns, particularly replacing 5,225ha of low profit rice by higher value crops such as vegetables, tea, maize and fruit crops.

In addition to the government support, there have been a number of assistance projects funded by donors, non-government organisations (NGOs) and the private sector. A number of projects have been reported by the surveyed respondents such as the system of rice intensification (SRI), an eco-friendly production method of water saving and greenhouse gas emission reduction (Truong et al., 2017), supported by Oxfam during 2011–2017 (Oxfam, 2011); reducing emissions from deforestation and forest degradation and enhancing forest carbon stocks (REDD+) in two pilot districts of Thai Nguyen since 2010 under the support UN REDD Facility (Pham et al., 2015); biogas development program during 2008–2015 with 8,186 biogas plants constructed in Thai Nguyen, mainly through two projects, namely, Biogas Program for the Animal Husbandry Sector in Vietnam, and Quality and Safety Enhancement for Agriculture Products and Biogas Plant (QSEAP) (UNEP-DTU, 2017). Also, Syngenta, a private company, has been reported to be an active actor through its introduction of new drought and disease resistant maize varieties in the studied areas since 2016. The new genetically modified (GM) varieties include NK4300BT/GT, DK6818S, and NK66BT/GT.

In short, there has been significant level of efforts from the local government and other actors in promoting CCA and response. Nonetheless, there has been limited information on local learning among farmers at the community level and their opinion of the most potential CCA initiatives for scaling. Nguyen et al. (2017b) found that the government policies mainly focus on “hard solutions” such as infrastructure improvement projects, while long-term CCA via ecosystem-based adaptation has not been paid with sufficient attention. Moreover, limited contribution budget from the local government is another hindering factor in realising the climate change adaptation and response in the province. According to a leader of the department of agriculture in Dinh Hoa district, the district could contribute to about 80% of the required contribution fund for the agriculture restructuring plan (2016–2020). In addition, the government's budget for agriculture extension was limited on small-scale pilot without further support for scaling. A leader of Vo Nhai department of agriculture stated similar situation of limited budget from the government that could only meet 30–40% of the needs and required tasks in agriculture and rural development in general, including CCA and response. The results were consistent with Ylipaa et al. (2019) who found that lack of government budget is the main issue that hampers the implementation of CCA efforts at the provincial and district levels. These authors also suggested that CCA efforts should not only rely on economic instruments and technical solutions from the government. This would imply a need for seeking and embracing local initiatives in the CCA efforts. Furthermore, the National Strategy on Climate Change (Decision 2139/QD-TTg (2011)) also emphasizes the need to build “communities which can effectively cope with climate change”. However, no evidence of efforts toward this model was identified during the survey in the province.

### 3.4 Potential climate resilient livelihood models and practices

Within the scope of this paper, some examples of climate resilient livelihoods in one district are presented. In-depth economic analysis and benefits of all the potential models and practices in the two districts are discussed in another paper.

A group discussion with the local farmers in Dinh Hoa district showed that the water saving techniques using sprinklers and mulching materials could save up to 60–70% of irrigation water compared to the traditional method using pumps without mulching. Also, their labour input for weeding was reduced by 40%. Fertiliser input was reduced by 10–15% due to the use of agricultural by-products as mulching materials. Hong and Yabe (2017) found that the traditional irrigation method in tea production in Thai Nguyen has rather low water use efficiency at 42.19%. Soil and water conservation practice was also reported to have positive impact on water use efficiency.

The system of rice intensification (SRI) has widely studied and reported with a number of benefits, including water saving, reduced inputs and labour, carbon footprint, while increased yield and income for rice farmers (e.g. Kassam et al., 2011; Thakur et al., 2016; Truong et al., 2017). This practice could be seen one of the potential climate resilient production practices in the studied areas where the majority of farmers practice rice-based production systems.

Wamatsembe et al. (2017) found that major constraints in maize production are drought and stem borers which would cause yield losses up to 54.7% and 23.5%, respectively. The use of genetically modified (GM) maize varieties could reduce yield losses and thus guarantee margin for farmers. According to CropLife Vietnam (2018), GM maize cultivar “NK4300 Bt/GT” has higher yield than non-GM variety “NK4300” of 15% in a normal condition in Thai Nguyen. Moreover, the GM maize has high drought tolerant and stem-borer resistant capacity. The focus group discussions among farmers and local organisations confirmed these characteristics and their intension to continue growing the MG maize cultivars to adapt to the increasing water shortage and pest manifestation. Although there have been some concerns and uncertainties with regard to possible negative impacts of GM crops on the environment, such impacts are still inconclusive (Dunfield and Germida, 2004; Fischer et al., 2015). Additionally, evident positive impacts on reduction of poverty and increased yields and income have been reported, leading to wide adoption of GM crops in various parts of the world (Brookes and Barfoot, 2014; Qaim and Kouser, 2013). From a holistic view on agricultural systems also the strong correlation with increased use of herbicides and dependencies from multinational companies should be considered.

A recent shift from one-crop rice land to grow other crops such as tea, fruit crops and medicinal plants in the research areas could be regarded rational for two reasons. According to the key informant interviews, tea and fruit crops are much higher value crops compared to rice. In addition, the crop conversion is suitable with the increasing water shortage in dry seasons. This is also in alignment with the central and local governments’ agricultural restructuring plans (Decision No. 1819/QD-TTg (2017) of the PM on approval for the agriculture restructuring plan for the period 2017–2020). Conversion of low-profit rice land to other commercially viable and productive crops or aquaculture is guided in the restructuring plan for implementation at localities (ASEM, 2018).

Despite the recent losses of farmers due to African swine fever, the integrated production model that combines animal husbandry and a biogas digester for treating farmyard manure was still regarded a potential climate resilient production model. Ha (2007) and Ha (2008) uncovered multiple benefits of this model, including improved rural environment, increased crop and fish yields, and farmer income. The use of bio-slurry as a clean source of organic fertiliser helps improve soil fertility. Additionally, the farming households can use biogas for cooking and lighting (energy saving effects).

### 3.5 System dynamic modelling for defining strategic actions toward sustainable livelihoods and income

**Figure 5:**
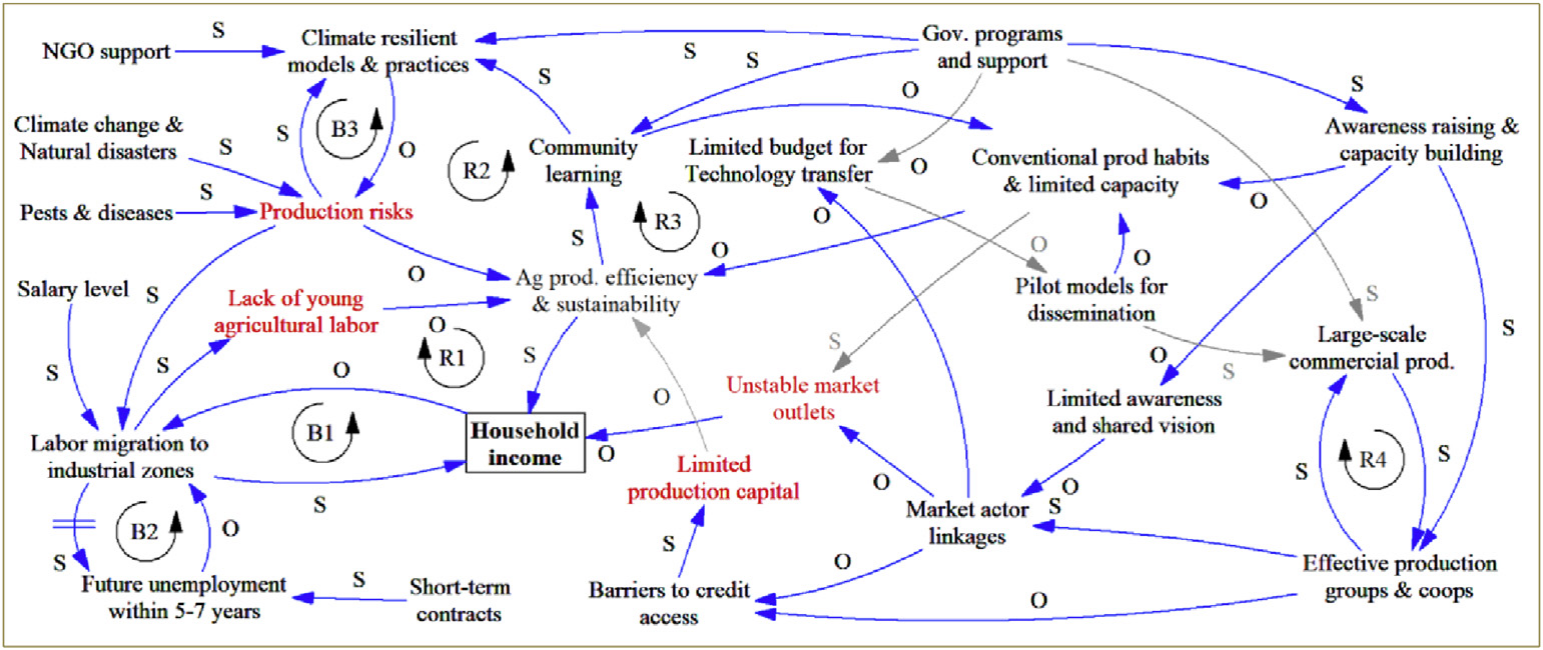
System dynamic model of the current situation of livelihoods of smallholder farmers in Thai Nguyen. *Legend: S - same direction; O - opposite direction; R - reinforcing (loop); B – Balancing (loop). Red variables represent key challenges faced by the farmers.*

Results from Steps 1 & 2 showed similar **challenges of smallholder farmers** in both districts, namely, unstable market outlets, production risks, lack of young labourers, limited production capital (red texts in Figure 5). *Insecure market outlets* were stated the most prominent challenge among farmers in both districts. According to Ha et al. (2016), unstable market outlets and low prices are mainly due to the small-scale production, ineffective producer groups and lack of formal contractual agreements with agribusinesses. *Production risks* were said to be caused by increasing impacts of climate change and natural disasters, and pests and diseases. The increased production risks together with low profitability of agricultural production have recently led to increasing number of young family members seeking off-farm jobs, particularly in industrial zones in Thai Nguyen and adjacent provinces. Accordingly, *shortage of young labourers* was stated as a significant challenge in agricultural production in the studied areas. This is consistent with findings of Ngoc and Yokoyama (2019) and Justina and Jonas (2008) who found both production risks due to harsh environments and low profitability of agricultural production as the two major causes of labour migration for off-farm job opportunities to meet their basic needs and income improvement. Finally, *limited production capital* was asserted due to limited financial resource and barriers to credit access. This challenge was reported as an inherent nature of smallholder farmers in Vietnam, particularly the rural poor in the northern midland and mountainous region (Ha et al., 2017).

Figure 5 presents a “big picture” of the current situation of local farming households, in which the key challenges (red variables) are influenced by various factors within the system that together affect production efficiency and sustainability, and eventually household income. The poor income situation induced a significant proportion of migrant work in industrial zones and other off-farm seasonal work in order to meet their immediate basic needs and earn additional income for their families (balancing loop B1, Figure 5). Nonetheless, the migration of young family members was said to have considerable impact on agricultural production efficiency and sustainability, which in turn influences their income from agricultural production (reinforcing loop R1, Figure 5). Findings of this study supports the view of Ngoc and Yokoyama (2019) who affirmed that migrant work is regarded a “short-term” solution, while waiting for a more viable and long-term agricultural resolution. Leaders of the two districts also acknowledged the future unemployment of the non-skill labourers in industrial zones within the next 5–7 years due to short-term contracts. Thus, this income stream would be considered a “quick fix” and/or temporary solution since the future unintended impact is foreseen (B2, Figure 5). According to the workshop participants, more support from the local government in training skilled labour for more secure and sustainable off-farm jobs are recommended.

Climate-smart production models and practices have been regarded potential adaptation initiatives in other parts of Vietnam (e.g. Grosjean et al., 2016; Nguyen et al., 2017a; Son et al., 2019; Tariq et al., 2018; Tran et al., 2016). The identified potential livelihood models and practices (Table 5) could be seen a rational efforts from the local government, NGOs and international programs (e.g. Oxfam America, and REDD + program) together with local initiatives. The production risks caused by climate change and natural disasters has triggered adoption of climate resilient production models and practices in order to mitigate and/or adapt with the advert impact of climate change (B3, Figure 5). Community learning was defined as one of the important interventions that could promote wider adoption of climate resilient practices, while it helps build capacity among community members and raise awareness of the unsustainable production practices (R2, R3, Figure 5).

**Table 5:** Examples of climate resilient livelihoods/production practices in Dinh Hoa district.

#	Potential production models/practice	Reasons for adoption/key benefits
1	Water saving techniques for tea production (using sprinklers and mulching materials).	Saving irrigation water and energy; Reduced labour input for weeding; Utilising agricultural by-products as mulching materials that help keeping soil moisture, and supplement nutrients for the plants. Thus, reduced fertiliser input.
2	System of rice intensification (SRI) and/or Alternate Wetting and Drying (AWD) method in rice production.	Reduced water usage; Improved yield; Stronger plant growth and reduced pests and diseases.
3	Use of drought and disease resistant maize varieties (GM maize).	Drought tolerance and diseases resistance; Improved yield.
4	Planting fruit crops on one-crop rice land areas.	Improved income, while adapting to the current context of increasing water shortage and drought.
5	Animal husbandry (pig, cattle) combined with Biogas digester installation.	Treating farmyard manure to become clean organic fertilizers; Reduced air pollution; Utilising energy (biogas) for cooking.
6	Use of biological pad (buffer materials mixed with beneficial microorganisms for deodorising odour and decomposing muck) in animal husbandry.	Reduced air pollution; Treating muck to become organic compost.

Government support was also regarded essential in promoting community learning, capacity building and awareness raising for farmers, technology transfer of improved production models, and promote large-scale production in accordance with building effective production groups and cooperatives. Its recent policies on promoting investment of the private sector in agriculture, market linkage strengthening and land consolidation for large-scale production (Resolution 05/2019/NQ-HDND of Thai Nguyen People's Committee on 23 July 2019) would contribute to the enhancement of market actor linkages in addressing the current challenges of local farmers (i.e. unstable market outlets and limited production capital) (Figure 5). According to Ha et al. (2015), improved management capacity of producer group and cooperatives enables economy of scale, while improving bargaining power, market actor linkages, and reducing barriers to credit access.

**Figure 6:**
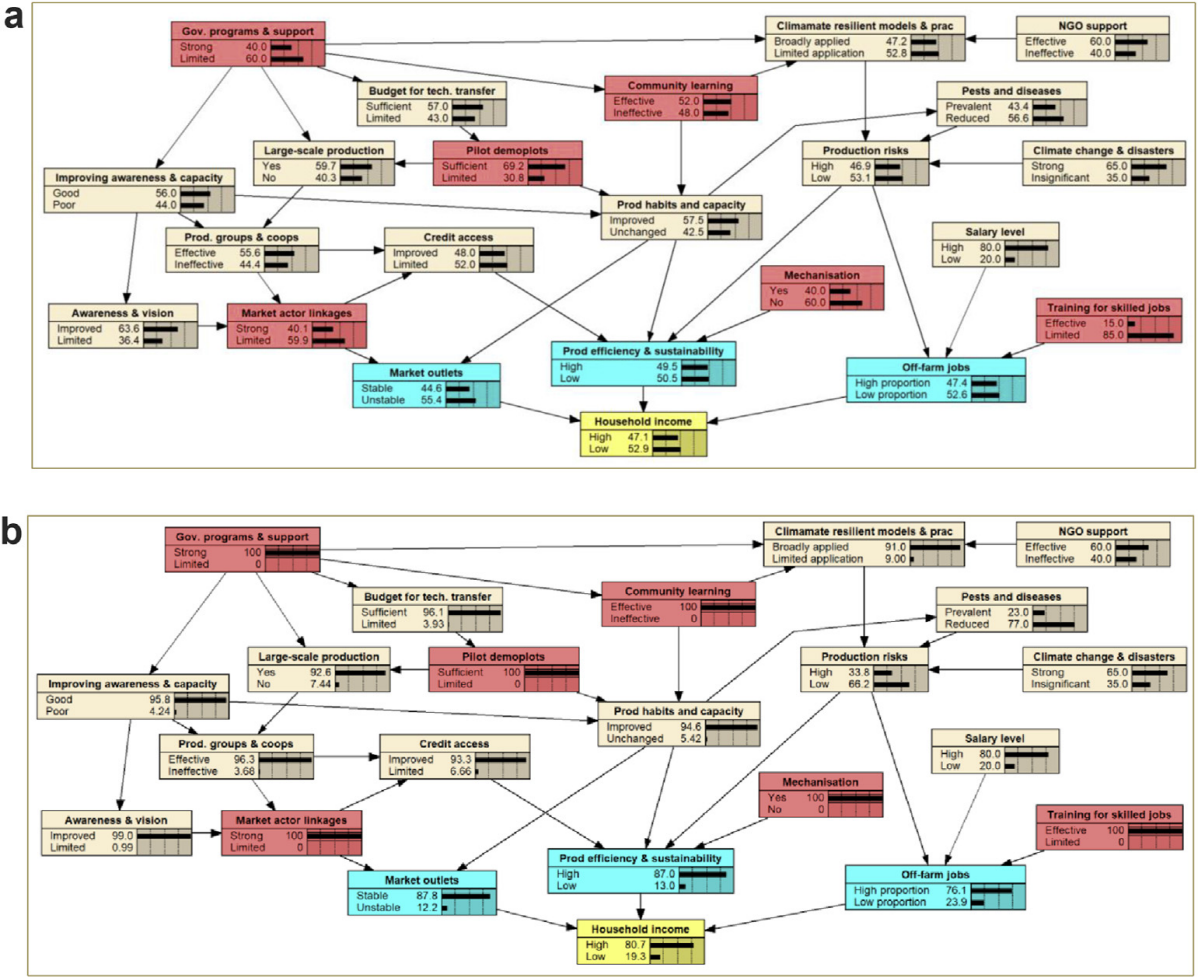
aBBN model of the current situation of farming households in Thai Nguyen province. *Notes: red colour nodes represent systemic interventions*; b: BBN modelling for identifying systemic interventions to improve income of the farming households in Thai Nguyen.

The key variables and/or factors that influence the livelihoods and income of local farmers were combined into one Bayesian Belief Network (BBN) model which reflects the current situation of the farming households in Thai Nguyen (Figure 6a). Household income was determined as the final goal to be achieved by the farmers through obtaining three “leverage points”, namely, more secure market outlets, improved production efficiency and sustainability, and off-farm jobs. Changes in these variables have high influences on the household income level. Of those, market outlets have the highest impact, followed by production efficiency and off-farm jobs. It is worth noting that off-farm jobs were referred to more skilled jobs through training rather than non-skill seasonal work and/or short-term and insecure jobs in industrial zones. The probability that their income is high is currently at only 47.1% (Figure 6a).

Testing the effects of various factors in the model on “household income” revealed a number of “**systemic interventions**” that are indicated in red colour nodes (including government programs and support; market actor linkages; pilot demo-plots; community learning; mechanisation in production; and training for skills jobs) (Figure 6a). The combination of all identified systemic interventions shows a significant increase in the probability of a higher household income (80.7%) of the smallholder farmers in Thai Nguyen (Figure 6b).

Results from this study suggest a need for a more holistic approach for synchronous support toward improving livelihoods, income and eventually the quality of life of the smallholder farmers. Given the context in Vietnam, the government support remains essential in agricultural development and poverty alleviation (Ha et al., 2016). However, for sustainability and scalability of the identified potential climate resilient models and practices, local learning and ownership would be regarded equally important. The importance of social learning and/or community learning in embracing climate-smart agriculture toward sustainability has been emphasized in a number of previous studies (e.g. Ensor and Harvey, 2015; Shaw and Kristjanson, 2014; Tran et al., 2017).

Based on the analyses and workshop discussions, the following strategies were proposed in order to achieve the final goal:

• Improve market access through improving market actor linkages with an important role of the local government to raise awareness and promote large-scale production and more organised and effective cooperatives.
• Improve production efficiency and sustainability with strong focuses on effective pilot implementation of field demo-plots together with promotion of community learning on the climate resilient models and practices, and support of mechanisation. Budget for agricultural and rural development programs such as the national target program on new rural development, poverty reduction, and agricultural restructuring agenda of central and local governments as well as non-government organisations could be utilised.
• Provide support for off-farm income generation activities through effective and relevant training for skills jobs.

The developed strategies would not only be relevant for the studied area, but could also be adopted in other regions for smallholder farmers in other parts of the world. This is because of similar challenges and characteristics of smallholder farmers in terms of market access challenges (e.g. Barrett et al., 2012; Biénabe and Sautier, 2005; Fischer and Qaim, 2012; Ha et al., 2015; Hazell and Rahman, 2014); production risks, particularly under the context of climate change (Eakin, 2000; Harvey et al., 2014; Lasco et al., 2014); and the need for off-farm jobs to address their basic needs and income diversification (Babatunde et al., 2010; Beyene, 2008; Demissie and Legesse, 2013).

## 4 Conclusions

This study has provided in-depth analyses and insights of the vulnerable situation of smallholder farmers in rural areas of a northern midland and mountainous province in Vietnam under the context of changing environment. There is still a high proportion (20.3%) of households being classified as marginally poor and poor. Their limited financial resources, small-scale production in accordance with the subsistence and semi-commercial farming systems clearly reflect their limited capability to adapt with the increasing impacts of climate change. Seeking temporary work industrial zones could be seen a “short-term” and/or “quick fix” solution to address their basic needs and create an additional income stream for their families. There is a strong need for strategic support and/or initiatives in defining long-term solutions for the vulnerable communities.

Although there have been a considerable level of efforts by the central and local governments and other actors to help mitigate the impacts of climate change, there are evident shortcomings due to inadequate approaches and limited level of support. The tendency of too much focus on “hard solutions” (e.g. infrastructure projects) (Nguyen et al., 2017b), “top-down” planning and management policies on agriculture and rural development, and ineffective extension services in the northern mountainous region (Ha et al., 2017; Yen et al., 2013), whereas local initiatives, community learning and ownership seem to be neglected. Findings of this study have revealed a high need for fostering community and/or social learning to sustain and scale the identified climate resilient livelihood models and production practices. It also provided insights for the government and extension services to improve their approach in CCA support. It is therefore highly recommended that community and/or social learning for CCA should be mainstreamed into socio-economic development planning. Additionally, the identified locally appropriate CCA initiatives shall be embraced and replicated for wider impact and sustainability of farming systems under the context of climate change.

The key challenges of the local farmers were found to be multidimensional that are influenced by many factors. The challenges include unstable market outlets, production risks, lack of young labourers, and limited production capital. Climate change related risks are just part of many difficulties the farmers are facing. The systems approach helped to uncover the patterns of interrelationships among various factors that influence the lives of the rural farmers. It also enabled the community members and related stakeholders to identify informed strategies and systemic interventions in addressing their identified challenges.

This research has proven the effectiveness and validity of systems approaches and tools in structuring and solving complex issues in agricultural systems research and development. Particularly, this study supports the viewpoints of Wals and Schwarzin (2012) and Hertel and Rosch (2010) who acknowledged the need for embracing systems approaches and employing simulation tools to deal with complex issues of climate change adaptation in a sustainable way. A holistic approach is clearly required to unravel such complexity nature of the system in which the farmers are operating. Community learning for adoption and scaling of climate resilient production models and practices are just part of the systemic interventions that need to be implemented in a coordinated manner towards a more resilient future of the farming communities. Besides, this study also supports the view of Funk et al. (2020) and Bryan et al. (2013) regarding the need to recognise and employ indigenous and/or local adaptation initiatives that are locally appropriate and affordable for smallholder farmers.

## Declarations

### Author contribution statement

T. M. Ha: Conceived and designed the experiments; Performed the experiments; Analyzed and interpreted the data; Contributed reagents, materials, analysis tools or data; Wrote the paper.

I. Kuhling, D. Trautz: Analyzed and interpreted the data; Wrote the paper.

### Funding statement

This work was supported by the Vietnam National Foundation for Science and Technology Development (14/2019/TN).

### Data availability statement

The authors do not have permission to share data.

### Declaration of interests statement

The authors declare no conflict of interest.

### Additional information

No additional information is available for this paper.

## Appendix A Supplementary data

Annex 1_Supplementary material Annex 1_Supplementary material
