# Supplementary material for: A systems approach toward climate resilient livelihoods: A case study in Thai Nguyen province, Vietnam
Source: Heliyon. 2020 Nov 18;6(11):e05541. doi: 10.1016/j.heliyon.2020.e05541 (PMC7689166; doi:10.1016/j.heliyon.2020.e05541)
Supplement: Annex 1_Supplementary material [file mmc1.docx]

***Annex 1:*** Summary of key government responses to climate change related to the Agriculture, Forestry and Fishery sector.

| # | Government actions | Priorities related to Agriculture, Forestry & Fishery sector |
| --- | --- | --- |
| 1 | National strategy for natural disaster prevention, response and mitigation to 2020 (Decision 172/2007/QD –TTg by the Prime Minister (PM). | Disaster prevention, response and mitigation shall be integrated into socioeconomic development master planning and plans of every region, sector, and nation-wide. |
| 2 | National Target Program to Respond to Climate Change (Decision No. 158/2008/QD-TTg by the PM. | Mainstream climate change issues into socio-economic, sectoral and local development strategies, plans and planning. |
| 3 | Action Plan Framework for Adaptation and Mitigation of Climate Change of the Agriculture and Rural Development Sector for the Period 2008-2020 (Decision No. 2730/QD-BNN-KHCN). | Ensure the stable agriculture production and food security with the stable area of 3.8 million ha of two seasonal rice crops; Ensure safety of dyke and infrastructure systems to meet requirements in disaster prevention and mitigation, etc. |
| 4 | Socio-Economic Development Plan (SEDP) for 2011–2015 (Resolution No. 10/2011/QH13 (2011) of the National Assembly (NA). | Stress actions to deal with climate change, increase forest coverage, maintain rice production area, and improve water supply coverage, etc. |
| 5 | National Strategy on Climate Change (Decision 2139/QD-TTg (2011) by the PM. | Guarantee food security and water resource; Protect and develop forests sustainably, increasing the absorption of greenhouse gases and preserving biodiversity; Reduce greenhouse gas emission; Promote climate smart agricultural production practices; and Building communities which can effectively cope with climate change, etc. |
| 6 | Law on Natural Disaster Prevention and Control (No. 33/2013/QH13 of the NA); and Decree No. 66/2014/ND-CP on Detailing and guiding a number of articles of the Law on Natural Disaster Prevention and Control. | Assign tasks for establishing provincial disaster risk management strategies and plans, and establishing provincial-level commanding committees for natural disaster prevention, control, and search and rescue. |
| 7 | SEDP for 2016-2020 (Resolution No. 142/2016/QH13 (2016) of the NA. | Actively respond to climate change, prevent natural disasters, and enhance natural resource management and environmental protection; protect and develop forests; protect water resources and ensure sufficient supply of water for production and consumption. |
| 8 | Decree No.02/2017/ND-CP (2017) of the PM on policies on assistance in agriculture production for revival of production of areas suffering from losses caused by natural disasters and epidemics. | Guide conditions for getting assistance and levels of immediate support for farming households that suffer from natural disasters and epidemics. |
| 9 | Decision No. 899/QD-TTg (2013) of the PM on approving the master plan “agricultural restructuring towards raising added values and sustainable development” | Reduce environmental footprint and promote sustainable agricultural production practices; Prevent and respond to natural disasters; Store water for irrigation, and use of water saving production techniques; Increase forest cover to 45% by 2020. |
| 10 | Decision No. 1819/QD-TTg (2017) of the PM on approval for the agriculture restructuring plan - period 2017 – 2020. | Respond to climate change and protect environment and ecosystem, reduce emission in agricultural production; achieve forest coverage of 42% by 2020; improve irrigation systems and natural disaster prevention and responses; promote changes in crop structure and use of crops suitable with the changing environment. |
